# Supplementary material for: The characteristics of host lipid body biogenesis during coral-dinoflagellate endosymbiosis
Source: PeerJ. 2021 Jun 23;9:e11652. doi: 10.7717/peerj.11652 (PMC8234918; doi:10.7717/peerj.11652)
Supplement: Supplemental Information 1 [file peerj-09-11652-s001.docx]

**Supplemental information**

**The characteristics of host lipid body biogenesis during coral-dinoflagellate endosymbiosis**

**Guidance – remove this box before submitting!**

Yellow callout boxes provide general notes. Please remove the yellow boxes before submitting. For full guidance see <https://peerj.com/about/author-instructions>

Blue highlighted example text should be replaced or removed with your own information.

| **DO**  –Use clear and grammatically correct English.  –Save as US Letter size format.  –Ensure line numbering is enabled.  –Align text LEFT.  –Ensure title, abstract, and author information matches what is entered online during submission. | **DO NOT**  –Embed ANY figures or tables in the text. Instead, upload a separate file for each on the file uploads page when submitting. Example – If you have 3 figures, then you will upload 3 figure files & be asked to add a figure title for each. See <https://peerj.com/about/author-instructions/#figures> for figure formats. |
| --- | --- |

Hung-Kai Chen^1^, Sabrina L. Rosset^1^ ,Li-Hsueh Wang^1,2^, and Chii-Shiarng Chen^1,2,3*^

^1^National Museum of Marine Biology and Aquarium, Pingtung, Taiwan

^2^Graduate Institute of Marine Biology, National Dong-Hwa University, Pingtung, Taiwan

^3^Department of Biological Science and Technology, I-Shou University, Kaohsiung, Taiwan

Corresponding Author:

Chii-Shiarng Chen

2 Houwan Road, Checheng, Pingtung 944, Taiwan.

Email address: cchen@nmmba.gov.tw


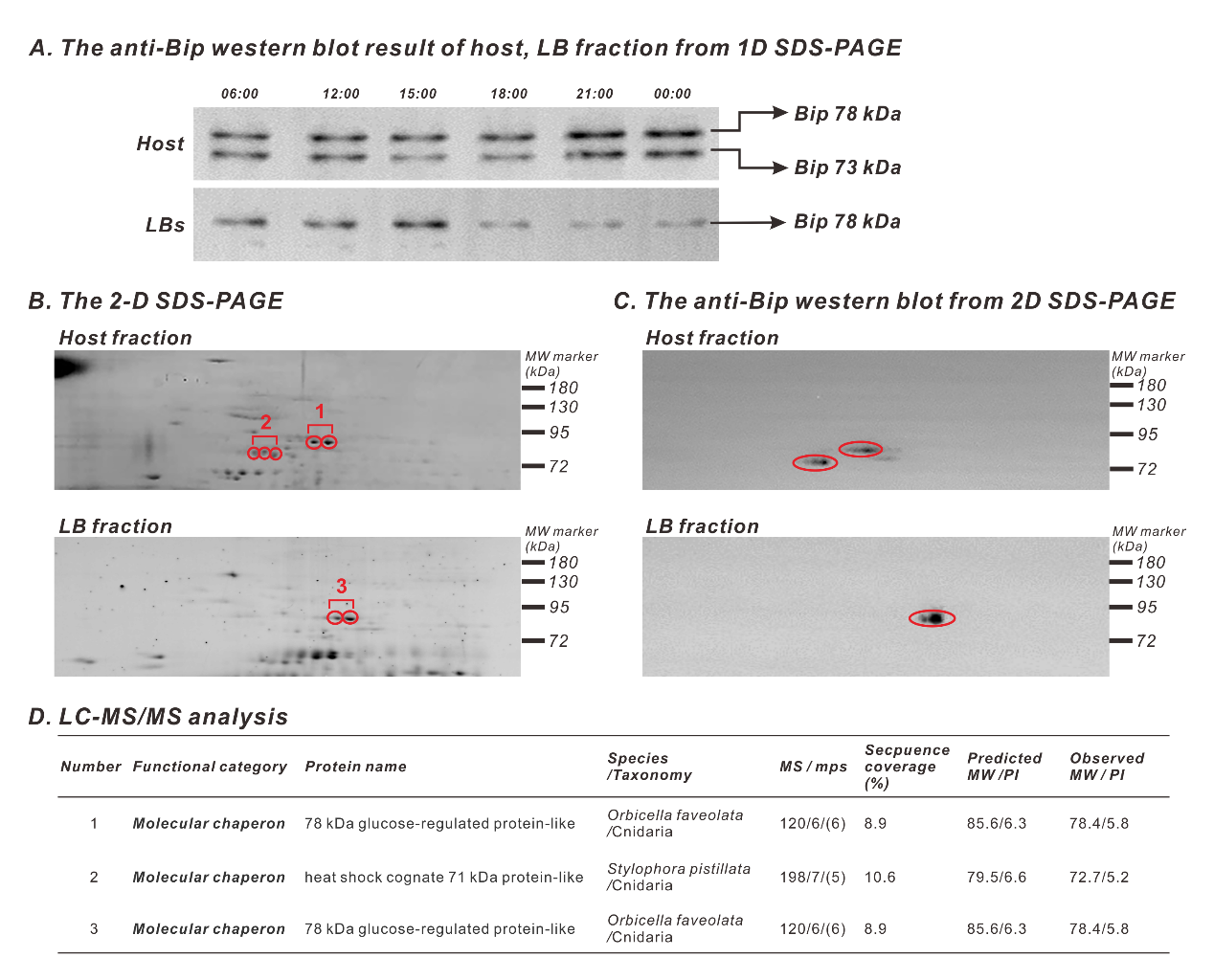


**Figure S1.** Western blot and LC-MS/MS analysis of BiP protein. Related to Figure 5. (A) The anti-BiP western blot result of host and LB fraction from 1D SDS-PAGE. (B) The 2-D SDS-PAGE of host and LB fraction. (C) The anti-BiP western blot result of host and LB fraction from 2D SDS-PAGE. (D) LC-MS/MS analysis.


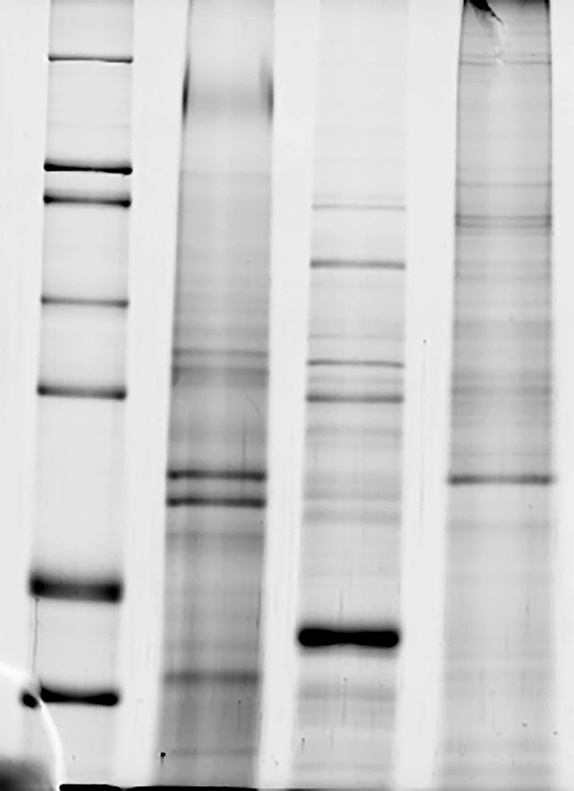

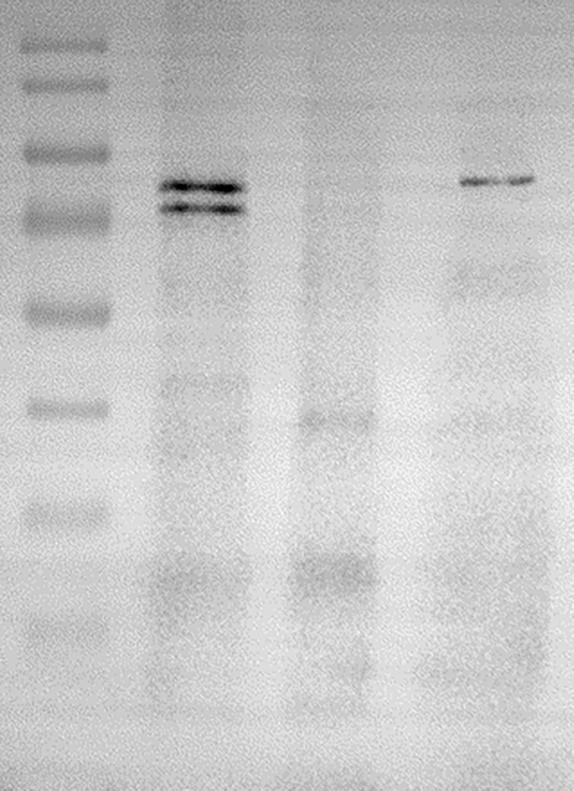
**A. B.**

**Figure S2.** Uncropped image of the anti-BiP purity analysis in different cell fractions. Related to Figure 5. (A) the SDS-PAGE result (B) the blot image (molecular maker, host cell, Symbiodinaceae and LB fractions from left to right lane, and 20 micrograms of each protein sample were subjected to analysis).


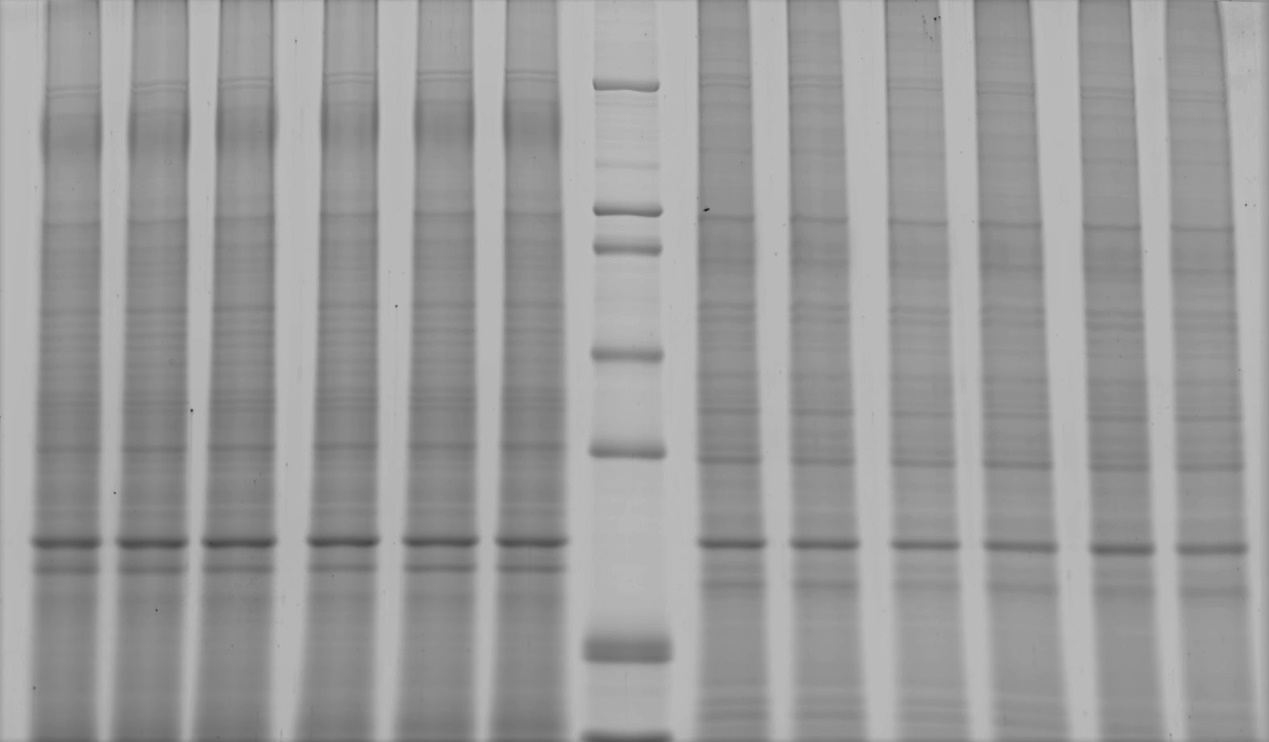
**A.**


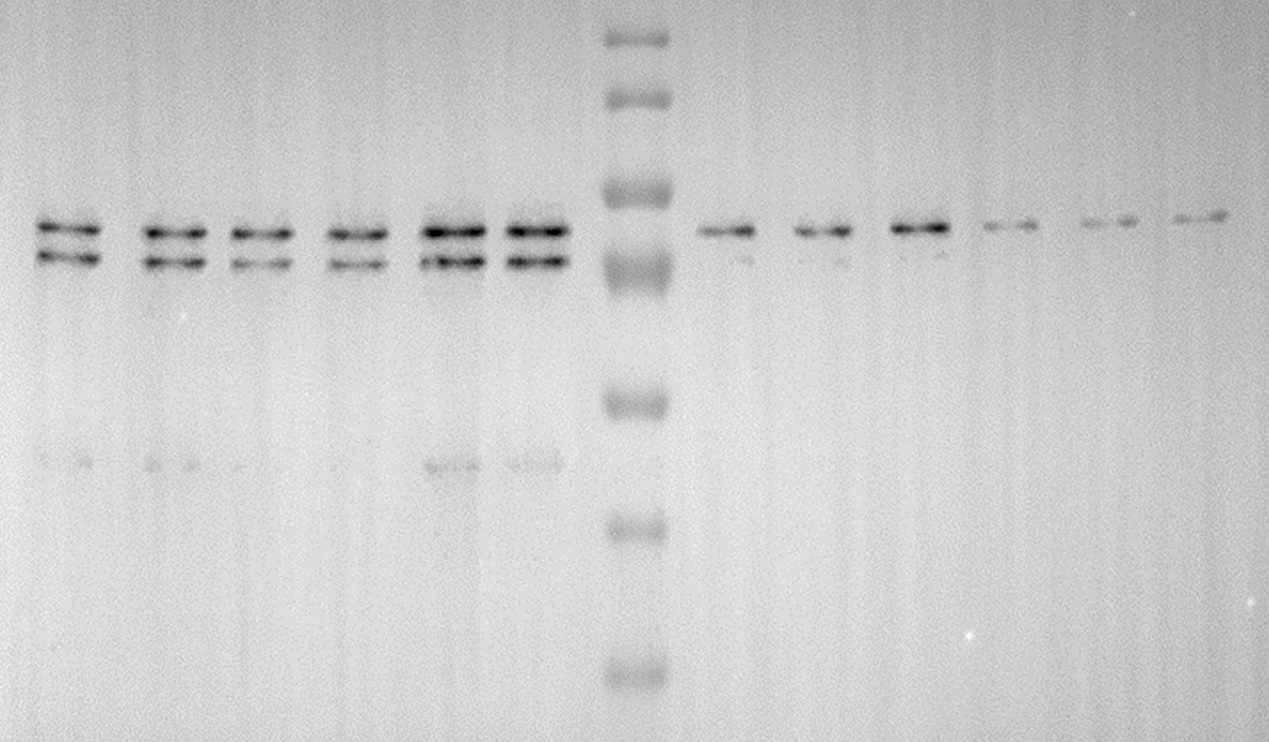
**B.**

**Figure S3.** Uncropped image of the SDS-PAGE and western blot analysis in host cell and LB fractions during diel cycle. Related to Figure 5. (A) the SDS-PAGE result (B) the blot image (host cell, molecular maker, and LB fractions from left to right lane, and 20 micrograms of each protein sample were subjected to analysis).


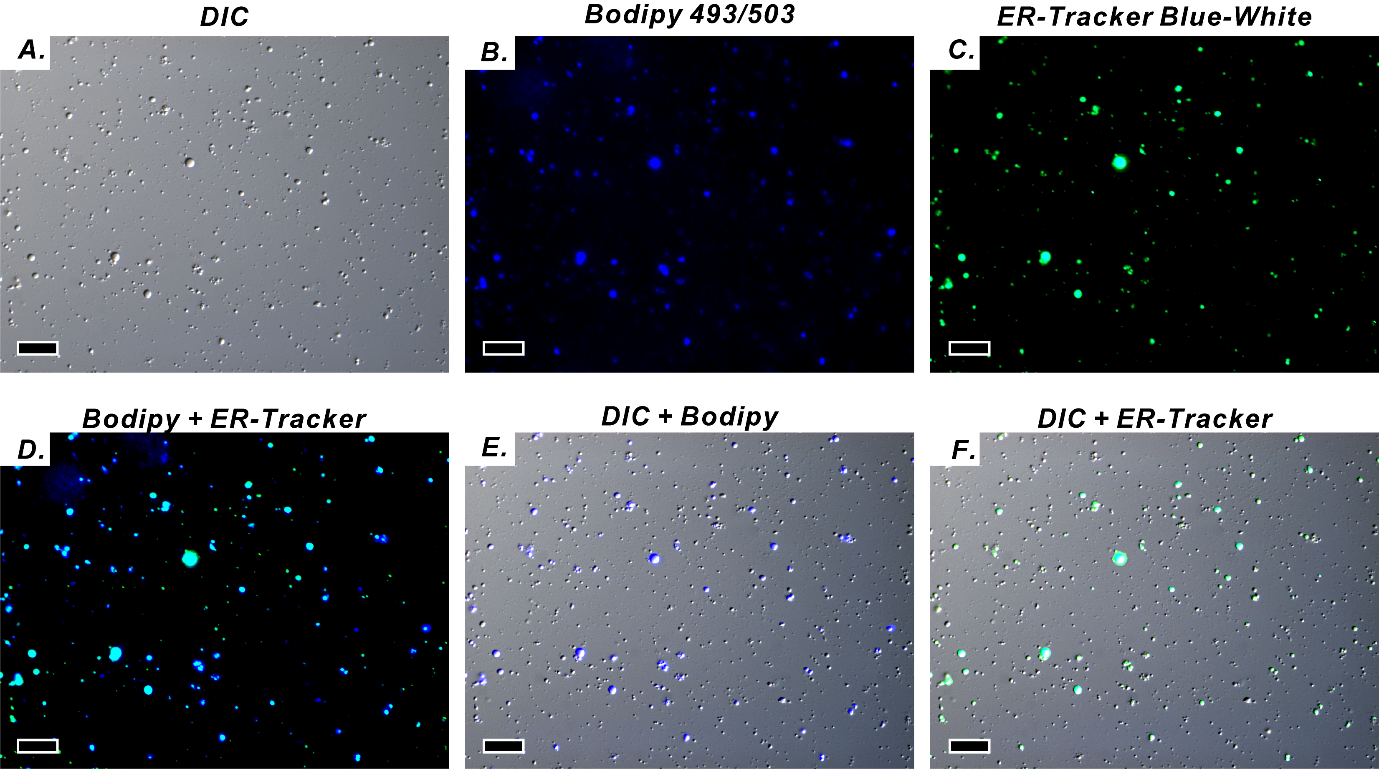


**Figure S4.** Merged image of the Lipid Bodies (LBs) purified from gastrodermal cells [A] under differential interference contrast microscopy (DIC). [B] LBs stained with the neutral lipid marker BODIPY 493/503. [C] LBs stained with endoplasmic reticulum (ER)-tracker Blue-White dye, which indicated that LBs were enclosed in an ER membrane. [D] Merged image of BODIPY 493/503 and ER-tracker Blue-White. [E] Merged image of DIC and BODIPY 493/503. [F] Merged image of DIC and ER-tracker Blue-White (scale = 10 μm).

**Table S1.** Different LB size and distribution during the diel cycle. Related to Figure 3. Data are presented as mean ± SD (n = 3), * *p* < 0.05, ** *p* < 0.01, *** *p* < 0.001

| Time | Total LB  Numbers | Distribution categories / % | **Micro-LB (<1μm)** | **Mid-LB**  **(1-3μm)** | **Large-LB (>3μm)** |  | **Total LB %** |
| --- | --- | --- | --- | --- | --- | --- | --- |
| 06:00  [Sunrise] | 153 ± 21.9^c^ | **Near mesoglea** | 41.0 ± 1.5^bc^ | 12.8 ± 2.1^a^ | 2.0 ± 0.6^a^ |  | 55.8 ± 2.1^ab^ |
|  |  | **Near coelenteron** | 22.3 ± 1.7^ab^ | 15.9 ± 0.4^b^ | 5.9 ± 2.4^bc^ |  | 44.2 ± 2.1^ab^ |
|  |  | F | 205.6 | 6.4 | 7.5 |  | 45.7 |
|  |  | *p* | *** | 0.065 | 0.052 |  | ** |
| 00:00 | 176.7 ± 17.2^c^ | **Near mesoglea** | 37.2 ± 2.9^c^ | 13.5 ± 4.4^a^ | 1.3 ± 0.4^a^ |  | 52.0 ± 2.8^b^ |
|  |  | **Near coelenteron** | 27.0 ± 2.5^a^ | 16.5 ± 2.2^ab^ | 4.4 ± 1.5^c^ |  | 48.0 ± 2.8^a^ |
|  |  | F | 20.9 | 1.2 | 12.7 |  | 3.2 |
|  |  | *p* | * | 0.344 | * |  | 0.149 |
| 12:00  [Noon] | 182.3 ± 12.1^c^ | **Near mesoglea** | 40.0 ± 1.8^c^ | 12.6 ± 1.9^a^ | 2.0 ± 0.5^a^ |  | 54.5 ± 3.7^ab^ |
|  |  | **Near coelenteron** | 20.3 ± 2.8^abc^ | 18.0 ± 2.5^ab^ | 7.2 ± 1.1^bc^ |  | 45.5 ± 3.7^ab^ |
|  |  | F | 106.8 | 8.7 | 57.1 |  | 9.1 |
|  |  | *p* | *** | * | ** |  | * |
| 15:00 | 309.7 ± 79.4^ab^ | **Near mesoglea** | 44.3 ± 3.8^abc^ | 10.2 ± 3.6^a^ | 0.9 ± 0.5^a^ |  | 55.4 ± 0.6^ab^ |
|  |  | **Near coelenteron** | 19.4 ± 5.0^abc^ | 17.5 ± 3.6^ab^ | 7.7 ± 1.7^bc^ |  | 44.6 ± 0.6^ab^ |
|  |  | F | 48.0 | 6.3 | 45.3 |  | 516.6 |
|  |  | *p* | ** | 0.066 | ** |  | *** |
| 18:30  [Sunset] | 319.7 ± 29.7^a^ | **Near mesoglea** | 40.1 ± 3.4^c^ | 13.2 ± 0.4^a^ | 1.3 ± 0.7^a^ |  | 54.6 ± 3.4^ab^ |
|  |  | **Near coelenteron** | 10.0 ± 3.1^d^ | 23.3 ± 1.1^a^ | 12.1 ± 0.7^b^ |  | 45.4 ± 3.4^ab^ |
|  |  | F | 127.3 | 216.6 | 349.6 |  | 10.6 |
|  |  | *p* | *** | *** | *** |  | * |
| 21:00 | 242.3 ± 30.1^abc^ | **Near mesoglea** | 44.7 ± 3.6^abc^ | 10.2 ± 3.2^a^ | 0.7 ± 0.6^a^ |  | 55.7 ± 4.4^ab^ |
|  |  | **Near coelenteron** | 13.8 ± 1.6^cd^ | 11.3 ± 4.9^b^ | 19.3 ± 1.7^a^ |  | 44.3 ± 4.4^ab^ |
|  |  | F | 181.3 | 0.1 | 330.4 |  | 9.8 |
|  |  | *p* | *** | 0.773 | *** |  | * |
| 00:00 [Midnight] | 212.3 ± 52.4^abc^ | **Near mesoglea** | 50.4 ± 6.6^ab^ | 11.2 ± 1.1^a^ | 1.1 ± 1.1^a^ |  | 62.7 ± 6.8^a^ |
|  |  | **Near coelenteron** | 18.3 ± 1.2^bcd^ | 11.4 ± 1.3^b^ | 7.6 ± 4.2^bc^ |  | 37.3 ± 6.8^b^ |
|  |  | F | 69.4 | 0.1 | 6.4 |  | 20.8 |
|  |  | *p* | ** | 0.837 | 0.065 |  | * |
| 03:00 | 203.7 ± 17^bc^ | **Near mesoglea** | 53.3 ± 2.8^a^ | 9.5 ± 1.1^a^ | 0.7 ± 0.2 ^a^ |  | 63.4 ± 3.1^a^ |
|  |  | **Near coelenteron** | 18.1 ± 1.9^bcd^ | 11.7 ± 1.0^b^ | 6.7 ± 2.6^bc^ |  | 36.6 ± 3.1^b^ |
|  |  | F | 329.3 | 7.0 | 16.5 |  | 113.0 |
|  |  | *p* | *** | 0.057 | * |  | *** |
| 05:00 | 162.7 ± 31.4^c^ | **Near mesoglea** | 50.7 ± 3.1^ab^ | 10.5 ± 2.3^a^ | 1.1 ± 0.5^a^ |  | 62.2 ± 2.0^a^ |
|  |  | **Near coelenteron** | 20.3 ± 4.2^abc^ | 13.0 ± 0.5^b^ | 4.5 ± 2.3^c^ |  | 37.8 ± 2.0^b^ |
|  |  | F | 102.4 | 3.3 | 6.6 |  | 216.2 |
|  |  | *p* | ** | 0.144 | 0.062 |  | *** |
| Significant level across time *(p)* | ***** | **Near mesoglea** | *** | 0.43 | 0.11 |  | ** |
|  |  | **Near coelenteron** | *** | *** | *** |  | ** |

**Table S2.** Unigenes related to ER stress expression level over the time. Related to Figure 4. The gene expression level of diel variation was calculated as relative expression values of each sampling time. We adopted log_2_(FPKM ratio) as the fold change of gene expression, that is, sunrise = log_2_(FPKM sunrise/midnight), noon = log_2_(FPKM noon/sunrise), sunset = log_2_(FPKM sunset/noon) and midnight = log_2_(FPKM midnight/sunset).

| **Name/ Time** | **FPKM** | | | |  | **Log_2_ (FPKM ratio)** | | | |
| --- | --- | --- | --- | --- | --- | --- | --- | --- | --- |
|  | **Sunrise** | **Noon** | **Sunset** | **Midnight** |  | **Sunrise** | **Noon** | **Sunset** | **Midnight** |
| BIP | 82.54 | 232.21 | 115.16 | 71.85 |  | 0.20 | 1.49 | -1.01 | -0.68 |
| ATF6 | 56.05 | 53.95 | 39.82 | 46.78 |  | 0.26 | -0.06 | -0.44 | 0.23 |
| S1P | 11.50 | 11.59 | 12.51 | 12.38 |  | -0.11 | 0.01 | 0.11 | -0.02 |
| IRE1 | 5.16 | 5.79 | 6.82 | 4.91 |  | 0.07 | 0.17 | 0.24 | -0.47 |
| XBP1 | 89.65 | 114.58 | 97.75 | 85.10 |  | 0.08 | 0.35 | -0.23 | -0.20 |
| PERK | 5.73 | 7.06 | 8.04 | 6.40 |  | -0.16 | 0.30 | 0.19 | -0.33 |
| eIF2α | 43.07 | 42.12 | 40.95 | 44.79 |  | -0.06 | -0.03 | -0.04 | 0.13 |
| SREBP1 | 22.67 | 25.60 | 24.42 | 23.69 |  | -0.06 | 0.18 | -0.07 | -0.04 |

**Table S3.** Percentage of ER related protein - BiP relative expression. Related to Figure 5D. Data are presented as mean ± SD (n = 3), * *p* < 0.05, ** *p* < 0.01, *** *p* < 0.001

| Time/ % | ***LB*** | ***Host*** |
| --- | --- | --- |
| ***06:30*** | 43.7 ± 0.02^a^ | 56.3 ± 0.02^b^ |
| ***12:00*** | 42.5 ± 0.07^a^ | 57.5 ± 0.07^b^ |
| ***15:00*** | 51.7 ± 0.10^a^ | 48.3 ± 0.10^b^ |
| ***17:30*** | 23.5 ± 0.05^b^ | 76.5 ± 0.05^a^ |
| ***21:00*** | 22.1 ± 0.04^b^ | 77.9 ± 0.04^a^ |
| ***00:00*** | 19.7 ± 0.02^b^ | 80.3 ± 0.02^a^ |
| Significant level  across time *(p)* | 17.7 | 17.7 |
|  | *** | *** |
